# Increased apelin receptor gene expression in the subfornical organ of spontaneously hypertensive rats

**DOI:** 10.1371/journal.pone.0231844

**Published:** 2020-04-21

**Authors:** Philip R. Griffiths, Stephen J. Lolait, Aarifah Bijabhai, Aoife O’Carroll-Lolait, Julian F. R. Paton, Anne-Marie O’Carroll

**Affiliations:** 1 Bristol Medical School, Faculty of Health Sciences, University of Bristol, Bristol, United Kingdom; 2 School of Medical Sciences, Faculty of Biology, Medicine and Health, University of Manchester, Manchester, United Kingdom; 3 Department of Physiology, Faculty of Medical & Health Sciences, University of Auckland, Auckland, New Zealand; 4 School of Physiology, Pharmacology and Neuroscience, Biomedical Sciences, University of Bristol, Bristol, United Kingdom; Max Delbruck Centrum fur Molekulare Medizin Berlin Buch, GERMANY

## Abstract

The vascular organ of the lamina terminalis, subfornical organ (SFO), and area postrema comprise the sensory circumventricular organs (CVO) which are central structures that lie outside the blood brain barrier and are thought to provide an interface between peripherally circulating signals and the brain through their projections to central autonomic structures. The SFO expresses mRNA for the G protein-coupled apelin receptor (APJ, gene name *aplnr)* and exogenous microinjection of the neuropeptide apelin (*apln)* to the SFO elicits a depressor effect. Here we investigated the expression and cellular distribution of *aplnr*, *apln* and the recently described ligand apela (*apela*) in the CVOs and investigated whether differences in the levels of expression of apelinergic gene transcripts in these regions might underlie the chronic elevated blood pressure seen in hypertension. We carried out multiplex *in situ* hybridization histochemistry on CVO tissue sections from spontaneously hypertensive rats (SHR) and normotensive Wistar Kyoto (WKY) controls. Confocal immunofluorescent images indicated strong *aplnr* expression, with lower levels of *apln* and modest *apela* expression, in the CVOs of both WKY rats and SHRs, in both neurons and glia. The expression level of *aplnr* transcripts was increased in the SFO of SHRs compared to WKY rats. Our data may highlight a potential dysfunction in the communication between CVOs and downstream signalling pathways in SHRs, which may contribute to its different phenotype/s.

## Introduction

Apelin, a 36 amino acid peptide, originally isolated from bovine stomach extracts, exists in numerous isoforms *in vivo*, all of which bind to, and activate, the G protein-coupled apelin receptor (APJ; gene name *aplnr*) [1, 2, 3]. Apelin (gene name *apln*) and APJ are ubiquitously expressed in peripheral cells and tissues, including vascular endothelial cells [4], cardiac myocytes [5], and kidney [6], as well as in discrete central regions such as forebrain (e.g. hypothalamic supraoptic (SON) and paraventricular (PVN) nuclei) and lower brainstem structures (e.g. nucleus of the solitary tract (NTS), area postrema (AP)) [7], thus allowing for multiple roles in the central regulation of physiological systems such as cardiovascular control [8, 9], angiogenesis [10], autonomic signalling [11–13], neuroprotection [14], fluid homeostasis [15], and modulation of hypothalamic-pituitary-adrenal axis activity [16, 17]. In some brain structures, e.g. cortex, APJ immunoreactivity (ir) is present in both neuronal and supporting cell populations such as glia [16]. Recently, an additional functional ligand for APJ has been identified, termed apela (gene name *apela*) or elabela [18–20], that is expressed in the rodent and human heart, kidney and placenta (e.g., see ncbi.nlm.nih.gov/gene/100506013; GEO Profiles). Apela is vital for normal cardiovascular development in embryonic zebrafish [18] and mice [21] and has been implicated in fluid homeostasis in adult rats through an action in the kidney [22]. Injection of apela into the cerebroventricular system is anorexigenic [23] although, to date, no endogenous central expression of apela has been reported [22].

A complex picture has emerged in the role that the apelinergic system plays in the modulation of blood pressure (BP), depending on the route of administration (systemic or central) and the brain region to which apelin is applied. The overall effect of systemic treatment with apelin-13 and [Pyr^1^]apelin-13, which may be acting at peripheral and/or central sites, is a depressor response in both anaesthetized rats [24–26] and conscious humans [27, 28] through increased vasodilatation and decreased vascular resistance. Additionally, intravenously administered apela normalized hypertension in pregnant apela knockout mice [21]. In contrast, central administration of apelin-13 and [Pyr^1^]apelin-13 directly to the ventricles [29] or to autonomic centres, such as the PVN [8] and rostral ventrolateral medulla (RVLM) [9, 30, 31], increases mean arterial BP via an increase in sympathetic nerve activity. Apelin may also act at other central sites to mediate cardiovascular responses. Interestingly, microinjection of apelin-13 to the subfornical organ (SFO) decreases BP and heart rate in anaesthetized rats [32], a response similar to that observed after systemic injection of apelin. The SFO, vascular organ of the lamina terminalis (OVLT), and the AP are collectively known as the sensory circumventricular organs (CVOs) and are midline, central structures that lie outside the blood brain barrier (BBB)—they have been identified as a possible interface between systemically circulating signals, such as ghrelin [33], leptin [34], vasopressin [35] and adrenomedullin [36], and the brain [37]. Efferent neurons from the CVOs project to brain regions involved in cardiovascular control such as the PVN, OVLT, RVLM, and NTS [38–40]. *Aplnr* expression is found in the SFO in transcriptomic studies (e.g. microarray analysis and RT-PCR [32]) while apelin immunoreactive fibres are observed in the SFO, OVLT, and AP [7]. The precise anatomical location of apelinergic gene expression in the CVOs has not been reported and to our knowledge no role as been assigned to these apelinergic neurones. The levels of apelinergic gene expression in some tissues however are altered in rodent models of hypertension, e.g. the cardiac apelinergic system is downregulated in the Dahl salt-sensitive rat [41], and decreased *aplnr* and *apln* levels are seen in the heart and aorta of spontaneously hypertensive rats (SHR) compared to normotensive Wistar Kyoto (WKY) rats [42], while APJ and apelin mRNA and protein are upregulated in the SHR PVN [8].

We hypothesized that changes in the levels of APJ, or its ligands, within the CVOs may lead to aberrant activity in the afferent arm and central circuitry of the autonomic nervous system manifesting in chronic elevated BP, or hypertension [43, 44]. Additionally, local apela gene expression in distinct brain regions involved in autonomic cardiovascular control would lend an intriguing layer of complexity to the role of the apelinergic system in the brain. Therefore, this study aimed to determine the anatomical localization of *aplnr*, *apln* and *apela* in distinct cell types in the SFO, OVLT, and AP, in normotensive rats using multiplex *in situ* hybridization histochemistry (ISHH), and to determine whether the hypertensive phenotype of SHRs is associated with differences in the levels of apelinergic gene expression in these regions.

## Materials and methods

### Animals

Adult male (approx 250g) Wistar Kyoto (WKY, Envigo, UK; n = 4) and spontaneously hypertensive rats (SHR, Animal Services Unit, University of Bristol, UK; n = 4) were used in this study. Animals were housed under constant temperature (21±2°C), light (lights on from 0700 to 1900h) and humidity (45–50%) regimens with food and water ad libitum. Animal care and maintenance were performed in accordance with the Animal Scientific Procedures Act (1986) United Kingdom and approved by the Bristol University Animal Welfare and Ethical Review body.

### Branched chain *in situ* hybridization histochemistry (ISHH)

Naïve adult WKY rats and SHRs were humanely killed between 0900–1100 using concussion to the cranium and immediately decapitated with a small animal guillotine. Brains were removed, frozen on powdered dry ice and stored desiccated at -80°C prior to processing. Brains were then sectioned (CM3050 S, Leica Microsystems) and the OVLT (Fig 28 Bregma +0.60mm), SFO (Fig 42 Bregma -1.08mm) and AP (Fig 148 Bregma -13.80mm) were identified with reference to the Rat Brain Atlas [45]. Twenty consecutive sections (16μm) were collected from the central portion of each structure. Sections were thaw-mounted onto SuperFrost Plus slides (Fisher Scientific, UK) and every fifth section was stained with toluidine blue (0.1%) as a histological reference. Sections were stored at -80°C for at least a week before use.

A branched chain *in situ* hybridization histochemistry (ISHH) assay (RNAscope Multiplex Fluorescence Assay kit; Bio-Techne) was used as described previously [6]. Briefly, sections were post-fixed in 4% PFA (pH 7.4), dehydrated in increasing concentrations of ethanol and treated for 30 minutes with RNAscope Protease IV solution. Sections were then incubated for 2 hours with proprietary probes, designed by Bio-Techne, for the following rat sequences: *apela* (GenBank Accession XM_008772035 (LOC100912649); bp147-1053; 12 Z probe pairs), *apln* (apelin; NM_031612; bp2-996; 20 Z probe pairs), *aplnr* (APJ; NM_031349; bp147-1053; 20 Z probe pairs), *rbfox* (NeuN; NM_00134498.2; bp16-1116; 15 Z probe pairs) and *gfap* (Gfap; NM_017009.2, bp1539-2534; 20 Z probe pairs). The specificity of the *aplnr*, *apln* and *apela* probes have been validated previously [6]. A combination of 3 probes at ratio 50:1:1 was used on any one section, as determined by the channel (C1-3) designated to each probe. The fluorescent colour for each probe (Alexa 488 (green), Atto 550 (orange) and Atto 647 (far red)) was dependent on the fluorescent colour module (Amp4 AltA-C) and channels (C1-3) used. Sections were counterstained with DAPI as per manufacturer’s instructions. Positive (rat polymerase (RNA) II (DNA directed) polypeptide A, transcript variant 1 (*POLR2A)*, peptidylprolyl isomerase (*PPIB)* and ubiquitin C (*UBC*); 3-plex probe set (ACD#320891) and negative (*DapB* (of Bacillus subtilis strain)) (ACD#320871)) probes purchased from Bio-Techne were included in all experiments. Experiments were repeated twice on different slide sets.

### Image capture and statistical analysis

Figures for RNAscope are representative of sections from four animals. Experimental groups of 4 were used based on past experiments detecting a statistically significant difference (*p*<0.05) between groups. This was confirmed by statistical power calculations. The fluorescence signal in the OVLT, SFO and AP was visualized using a x40 oil lens on a Leica SPE single channel confocal laser scanning microscope attached to a Leica DMi8 inverted epifluorescence microscope equipped with a Lecia DFC365FX monochrome digital camera and LAS X workstation (Wolfson Bioimaging Centre, University of Bristol). Images were captured using the LAS X software and Z stacks (Z step size = 0.5μm) were taken for all images. Raw image files were processed to generate composite images using the open access image analysis software, FIJI [46]. Automated image analysis (for dots/DAPI nuclei) was carried out using a custom plugin for FIJI provided by the Wolfson Bioimaging Facility at the University of Bristol. To obtain the percentage of positive cells, DAPI-labelled nuclei were segmented and numbers of round dots over DAPI nuclei were manually counted independently by two observers. Statistical analysis was carried out using GraphPad Prism software v7 (GraphPad). Data was tested for normality using a Kolmogorov-Smirnov test. Non-parametric variables are expressed as medians with interquartile ranges (IQR) and statistical significance was testing using the Mann-Whitney test. *p*<0.05 was considered statistically significant between groups.

## Results

### Apelinergic distribution in rat circumventricular organs (CVOs) of WKY rats and SHRs

**Fig 1:**
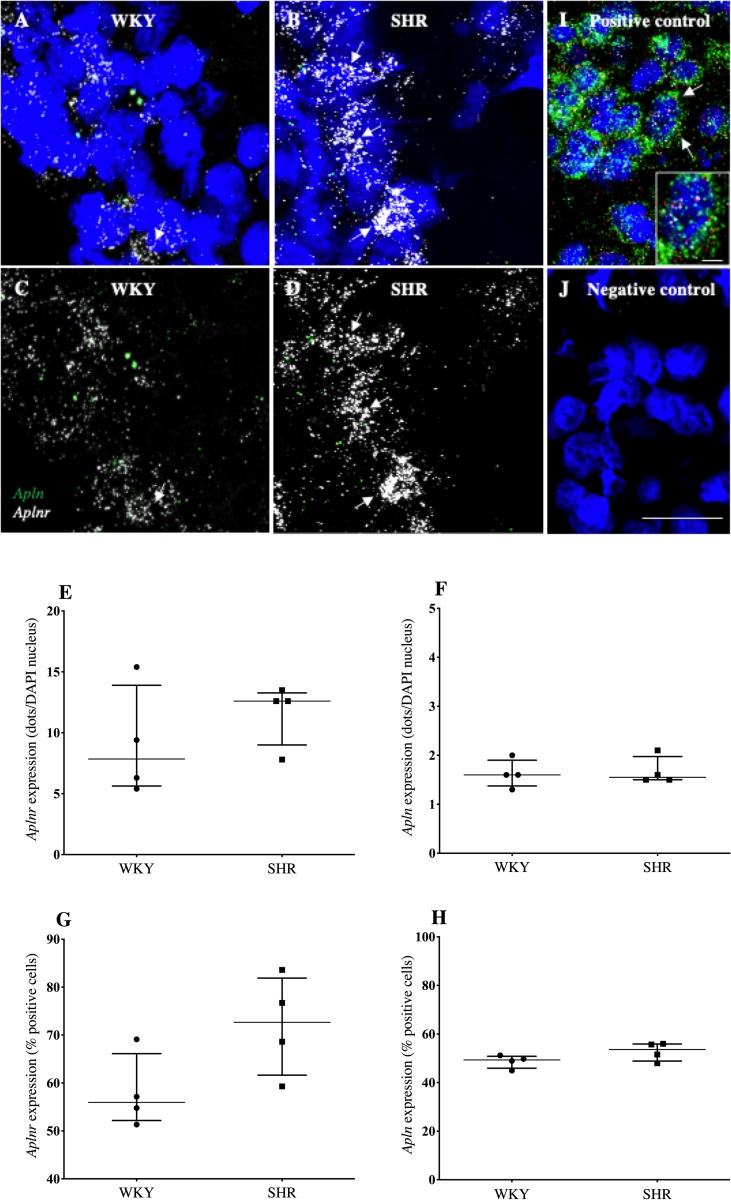
*Aplnr* and *apln* expression in the OVLT of WKY rats and SHRs. (A-D) Representative fluorescent images showing expression of *aplnr* and *apln* in the OVLT of WKY rats (A and C) and SHRs (B and D). DAPI: blue, *aplnr*: white, *apln*: green. DAPI removed from C and D to highlight distribution of mRNA. Arrows point to representative cells with relatively high levels of *aplnr* expression. (E-H) Graphs showing gene expression as a function of dots/DAPI nuclei or percentage positive cells for *aplnr* (E and F) and *apln* (G and H) expression. n = 4/group. Data is median [IQR]. Sections were also analysed with positive (rat polymerase (RNA) II (DNA directed) polypeptide A, transcript variant 1 (*POLR2A;*white dots), peptidylprolyl isomerase (*PPIB;* red dots) and ubiquitin C (*UBC*; green dots)) (I) and negative (*DapB* (of Bacillus subtilis strain)) (J) with nuclei counterstained with DAPI (blue). The inset shows a high magnification image of the cell depicted by white arrows. Scale bar = 50μm and 10μm for the inset image.

**Fig 2:**
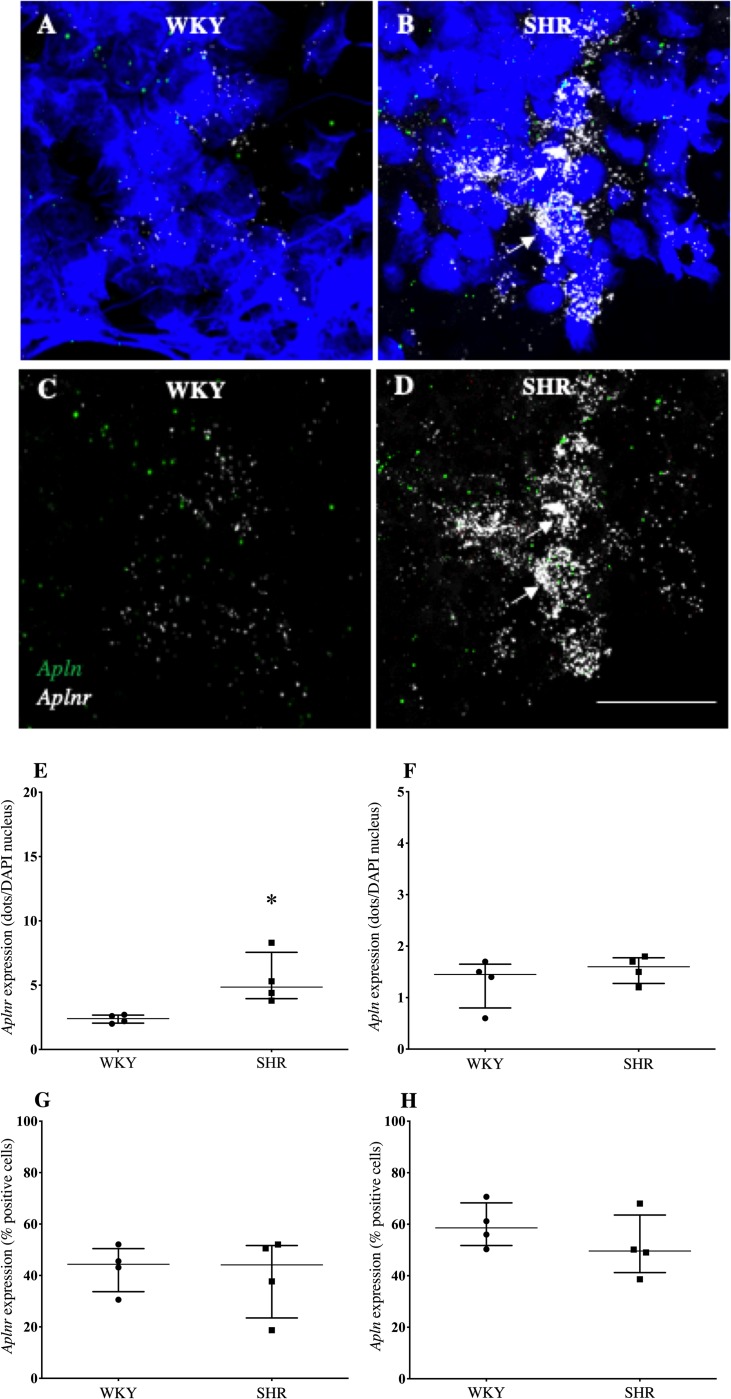
*Aplnr* and *apln* expression in the SFO of WKY rats and SHRs. (A-D) Representative fluorescent images showing expression of *aplnr* and *apln* in the SFO of WKY rats (A and C) and SHRs (B and D). DAPI: blue, *aplnr*: white, *apln*: green. DAPI removed from C and D to highlight distribution of mRNA. Arrows point to representative cells with relatively high levels of *aplnr* expression. Scale bar = 50μm. (E-H) Graphs showing gene expression as a function of dots/DAPI nuclei or percentage positive cells for *aplnr* (E and F) and *apln* (G and H) expression. n = 4/group. Data is median [IQR]. *p<0.05.

**Fig 3:**
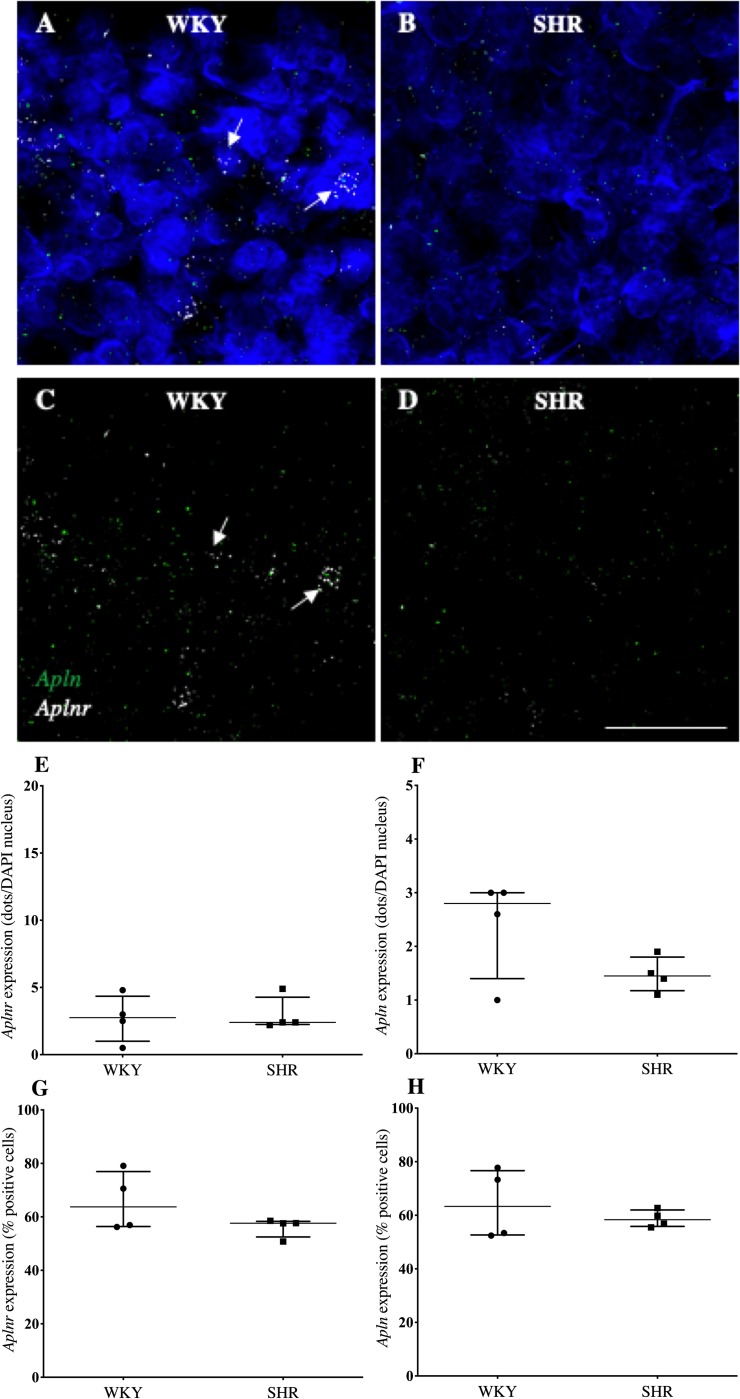
*Aplnr* and *apln* expression in the AP of WKY rats and SHRs. (A-D) Representative fluorescent images showing expression of *aplnr* and *apln* in the AP of WKY rats (A and C) and SHRs (B and D). DAPI: blue, *aplnr*: white, *apln*: green. DAPI removed from C and D to highlight distribution of mRNA. Cells with a high relative abundance of *aplnr* are arrowed. Scale bar = 50μm. (E-H) Graphs showing gene expression as a function of dots/DAPI nuclei or percentage positive cells for *aplnr* (E and F) and *apln* (G and H) mRNA. n = 4/group. Data is median [IQR].

**Fig 4:**
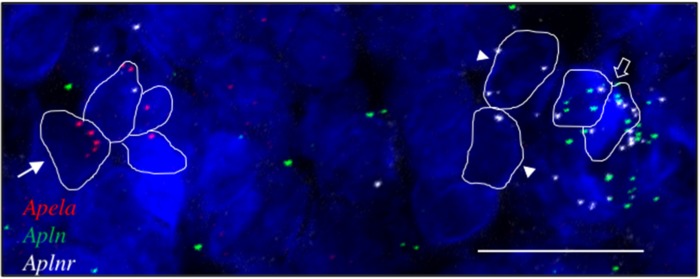
*Aplnr*, *apln* and *apela* transcripts in the SHR AP. Representative fluorescent images showing cells expressing *apela* (red dots; arrowed), *aplnr* (white dots, arrowheads) and *apln* (green dots). Cells co-expressing *aplnr* and *apln* are indicated (open arrow). Scale bar = 50μm. n = 4.

**Fig 5:**
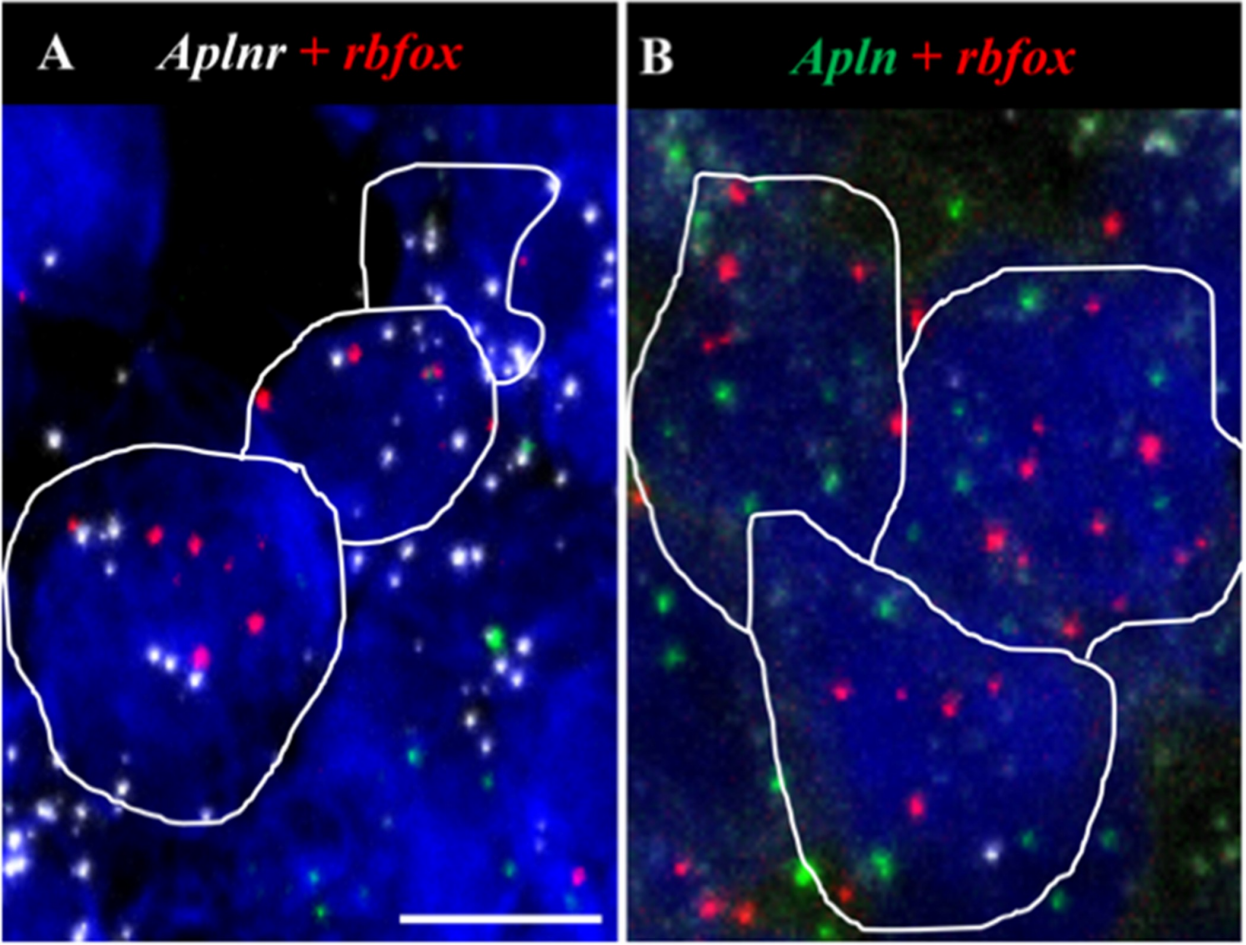
*Aplnr* and *apln* transcripts co-localise with *rbfox* in the AP. (A,B) Representative fluorescent images showing colocalization of *aplnr* (white dots, A) and *apln* (green dots, B) with *rbfox* (red) in the AP. Scale bar = 10μm. n = 4.

Confocal immunofluorescent images indicated *aplnr* was more abundantly expressed than *apln* in the OVLT (Fig 1), SFO (Fig 2) and AP (Fig 3) of both WKY rats (Figs 3A and 3C, 4A and 4C, 5A and 5C) and SHRs. (Figs 1B and 1D, 2B and 2D, 3B and 3D). *Aplnr* was relatively strongly expressed in all CVOs with a patchy distribution, in contrast to the level of *apln* expression which was low and more uniform. In the SFO and OVLT, *aplnr* expression was seen lateral of the main portion, and in the ventral portion of the lateral zone, respectively. Gene expression was quantified as a function of the number of dots/DAPI nuclei for each gene in both WKY rats and SHRs. The SFO from SHRs showed a significantly greater number of *aplnr* RNAscope dots/cell than normotensive WKY rats (4.9 [4.0–7.6] dots/cell vs 2.4 [2.0–2.7] dots/cell respectively; *p*<0.05, Fig 2E). No difference was seen in the number of *aplnr* RNAscope dots/cell in the OVLT or AP of SHRs (12.6 [9.0–13.2] dots/cell, Fig 1E; 2.4 [2.3–4.3] dots/cell, Fig 3E respectively) compared to WKY rats (7.9 [5.6–13.9] dots/cell, Fig 1E; 2.8 [1.0–4.4] dots/cell, Fig 3E respectively). Additionally, no difference was seen in the number of *apln* RNAscope dots/cell in the OVLT (1.6 [1.5–2.0] dots/cell, Fig 1G), SFO (1.6 [1.3–1.8] dots/cell, Fig 2G) or AP (1.5 [1.2–1.8] dots/cell, Fig 3G) of SHRs compared to WKY (1.6 [1.4–1.9] dots/cell, Fig 1G; 1.5 [0.8–1.7] dots/cell, Fig 2G; 2.8 [1.4–3.0] dots/cell, Fig 3G respectively) rats. The percentage of cells in each region positive for *aplnr* or *apln* gene expression was also calculated. There were no differences in the percentage of cells expressing *aplnr* (73 [62–82]%, Fig 1F) or *apln* (54 [–56]%, Fig 1H) in the SHR OVLT compared to WKY rat OVLT (56 [52–66]%, Fig 1F; 49 [46–51]% Fig 1H, respectively). Similarly there were no differences in the percentage of cells expressing *aplnr* or *apln* in the SHR SFO compared to WKY rats (44 [33–50]%, Fig 2F; 50 [41–64]% Fig 2H, respectively vs 44 [33–50]%, Fig 2F; 59 [52–68]%, Fig 2H respectively) or in the SHR AP compared to WKY rats (58 [56–62]%, Fig 3F; 58 [52–58]%, Fig 3H respectively vs 64 [57–77]%, Fig 3F; 63 [53–77]%, Fig 3H respectively). The increase in *aplnr* expression in SHR SFO appears to be in the amount of mRNA expressed per cell and not in the absolute number of cells expressing the gene.

Positive control probe sets detected mRNA throughout all sections (see Fig 1I) and gave the expected relative levels of gene expression with *UBC* (green) more abundant than *PPIB* (red) and *POLR2A* (white). The negative control probe set (*DapB*) generated only rare isolated signal (mainly in the far-red channel) (e.g. signal detected in one out of 89 cells; Fig 1J).

In a small follow up study, we investigated the expression of *apela* in the CVOs. *Apela* was much less abundantly expressed than *apln* (or *aplnr*) in all three CVOs, evident predominantly in the AP. Up to ~9% of cells in the AP expressed *apela* (9.4% (28/298 cells) and 5.5% (17/308 cells) of cells were *apela*-positive in two images of AP from different SHR rats (see Fig 4); 5.8% (16/276 cells) of cells were *apela*-positive in one image of AP from a WKY rat).

In contrast, in the SFO and the OVLT *apela* was rarely expressed and was seen in fewer than 1% of cells in either SHRs or WKY rats. Due to the low abundance of *apela* expression, we did not systematically determine whether *apela* was expressed in *rbfox*- or *gfap*-positive cells or differed between CVOs of SHRs or WKY rats.

### Localization of *aplnr* and *apln* to OVLT, SFO and AP neurons and/or glia in normotensive and SHR CVOs

**Fig 6:**
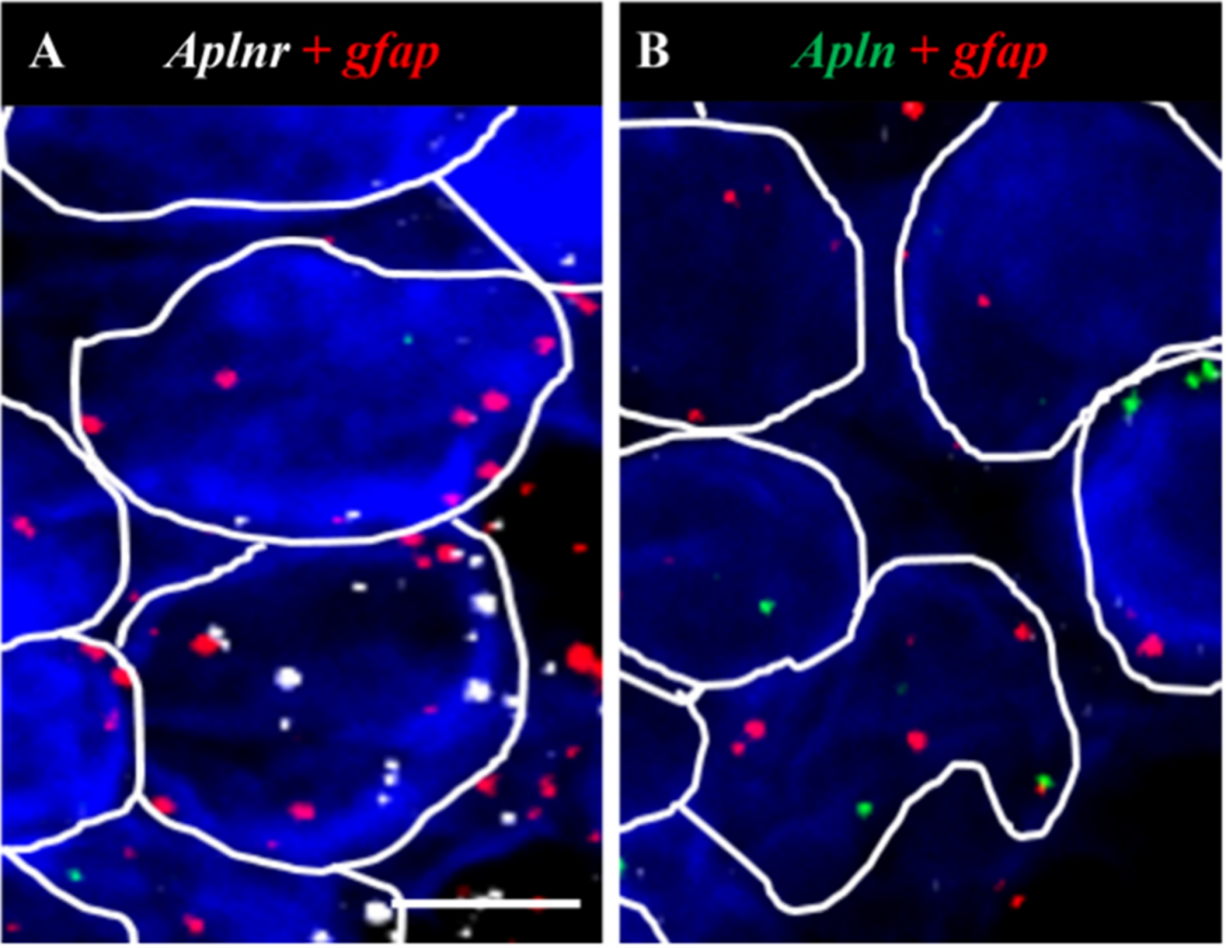
*Aplnr* and *apln* transcripts colocalise with *gfap* in the AP. (A, B) Representative fluorescent images showing colocalization of *aplnr* (white dots, A) and *apln* (green dots, B) with *gfap* (red) in the AP. Scale bar = 20μm. n = 4.

To examine the cell types expressing *aplnr* and *apln*, *aplnr*- or *apln*-positive cells were identified and the number of cells double-positive for either the neuronal marker *rbfox* (NeuN) or the prototypical glial-astrocyte marker *gfap* (Gfap) were quantified (4 brain slices/probe set). *Aplnr* and *apln* were expressed in both neurons and glial cells in all three CVOs in WKY rats and SHRs as evidenced by their colocalization with *rbfox* (e.g., Fig 5A and 5B respectively) or *gfap* (e.g., Fig 6A and 6B respectively).

The colocalization of both *aplnr* or *apln* with *rbfox* was decreased in the SHR AP (11 [8–13]% and 26 [24–27]% respectively, Table 1) compared to WKY rats (35 [29–50]% and 56 [48–71]% respectively (Table 1, *p*<0.05) while the co-localization of both *aplnr* or *apln* with *gfap* was increased in the SHR OVLT (76 [74–78]% and 49 [43–69]% respectively, Table 2) compared to WKY rats (51 [49–55]% and 29 [26–36]% respectively, Table 2, *p*<0.05). No other change in the co-localization of *aplnr* or *apln* with *rbfox* or *gfap* in the three CVOs was seen between SHRs and WKY rats (Tables 1 and 2 respectively).

**Table 1:** The percentage of *rbfox* positive cells co-localizing with *aplnr* or *apln* in the OVLT, SFO and AP of WKY rats and SHRs.

	% *rbfox* Positive cells					
	OVLT		SFO		AP	
	*Aplnr*	*Apln*	*Aplnr*	*Apln*	*Aplnr*	*Apln*
**WKY**	18[11–36]	39[30–45]	17[10–26]	53[39–75]	**35[29–50]**	**56[48–71]**
**SHR**	28[22–48]	38[24–60]	11[10–16]	43[23–58]	11[8–13]*	26[24–27]*

n = 4/group. Data is median [IQR].

*: *p<0.05*.

**Table 2:** The percentage of *gfap* positive cells co-localizing with *aplnr* or *apln* in the OVLT, SFO and AP of WKY rats and SHRs.

	% *gfap* Positive Cells					
	OVLT		SFO		AP	
	*Aplnr*	*Apln*	*Aplnr*	*Apln*	*Aplnr*	*Apln*
**WKY**	**51[49–55]**	**29[26–36]**	50[39–71]	44[38–58]	27[19–40]	31[22–39]
**SHR**	76[74–78]*	49[43–69]*	53[48–68]	34[27–49]	38[25–52]	43[30–51]

n = 4/group. Data is median [IQR].

*: *p<0.05*.

### Co-localization of *aplnr* and *apln* in normotensive and SHR CVOs

ISHH to detect *aplnr* and *apln* in the same sections revealed that a proportion (16–42%) of *aplnr*-positive cells in the three CVOs also expressed *apln*, however a number of cells also expressed either *aplnr* or *apln* alone. There was a significant decrease in the proportion of cells expressing both *aplnr* and *apln* in the AP of SHRs in comparison to WKY rats (24 [18–39]% vs 41 [32–47]% respectively, *p*<0.05). No change in the percentage co-localization of *aplnr* with *apln* was seen in the OVLT or SFO of SHRs in comparison to WKY rats (36 [29–40]% vs 31 [16–39]% and 15 [12–20]% vs 20 [17–26]% respectively, *p*>0.05).

## Discussion

The present study confirms that the apelinergic system in the sensory CVOs has a potential role in mediating cardiovascular responses. We employed highly specific RNAscope multiplex ISHH to demonstrate expression of *aplnr*, *apln* and *apela* transcripts in all three CVOs in both WKY rats and SHRs. Additionally, we report *aplnr* and *apln* were expressed in both neurons and glial cells in the three CVOs in these rat strains. Importantly *aplnr* levels within the SFO of SHRs were significantly higher compared with normotensive WKY rats, suggesting that this receptor within the SFO may be associated with the pathogenesis of hypertension.

APJ/apelin/apela have important roles in the regulation of cardiovascular function and control of BP as evidenced in numerous studies [8, 9, 20, 24, 29–31, 47–58]. Some of these central effects may be mediated by APJ in the SFO, OVLT and/or AP. The SFO is a mid-line sensory CVO that has been shown to project to the preoptic-hypothalamic circuit, OVLT, and brainstem cardiovascular centers such as the RVLM, while the OVLT itself projects to the PVN (a source of presympathetic neurons regulating sympathetic outflow via descending fibres) and SON; and the AP directly projects to the NTS, dorsal motor nucleus of the vagus, ventrolateral medulla and the lateral division of the parabrachial nucleus [59]. All three CVOs are known to be involved in the regulation of the cardiovascular system, fluid balance and energy homeostasis (see [59] for review).

Central structures, including the SFO, OVLT and brainstem structures involved in the modulation of BP, contain apelinergic ir-fibres [56] and express *aplnr* [17]—anatomical observations that support roles for apelin and APJ in the control of the cardiovascular system. By contrast *apela* expression has been found in the zebrafish and rodent cardiovascular system [6, 18, 19], however while expression has been demonstrated in developing and adult human and mouse brain samples in transcriptomic repositories (such as *Ensembl*; GEO Profiles), no anatomical distribution of *apela* in the CNS has been described in the literature. We report the expression of *apln*, *aplnr* and *apela* within the three CVOs in SHRs and WKY rats. Since apelin presumably does not cross the BBB (although this has been disputed [60], and may change in hypertension [61]), the CVOs are likely important conduits allowing circulating apelin access to the brain. These sites also incorporate a high degree of neural interconnectivity and reciprocal communication, which may thus transduce peripheral apelin signals in critical cardiovascular control centers that regulate sympathetic activity to maintain BP. The presence of *apln* in the CVOs indicates that apelin may be synthesised locally within these brain regions. While the major site of apelin/APJ expression in the brain appears to be the hypothalamus, apelin is detected in neuronal cell bodies and fibres throughout the entire brain [56]. Apelin is also found in the systemic circulation, possibly secreted from adipocytes or endothelial cells [62, 63]. Both systemic [64] and central [8, 30] administration of the APJ antagonist F13A abolishes apelin-mediated MABP effects. The co-existence of *aplnr* and *apln* (and to a lesser extent *apela*) in the CVOs, outside the BBB, indicates an intimate connection between the central and peripheral apelinergic systems and suggests that APJ within these organs may be activated by locally synthesised apelin (suggesting autocrine/paracrine actions within the CVOs) and/or by circulating levels of apelin—the presence of *aplnr* in the choroid plexus [24] is consistent with the latter premise. Additionally, the presence of *apela* expression within the CVOs raises questions as to which APJ ligand (and perhaps their proteoforms) is functionally active, on the same or different cells, during development and in response to which physiological perturbations.

Levels of peripheral apelinergic gene expression are downregulated during hypertension [41, 42, 58]. However, within the brain, we and others have shown APJ and apelin gene transcripts and protein are upregulated in the SHR PVN [8] and RVLM [9, 65] by mechanisms that are not yet understood. Here we report that *aplnr* levels within the SFO of SHR were significantly higher compared with normotensive WKY rats, highlighting a potential association of SFO *aplnr* with hypertension in SHRs, while there was no significant difference in *aplnr* levels in OVLT or AP, or in *apln* levels in any of the CVOs, between the two rat strains. The consistently increased *aplnr* expression observed in the SFO of hypertensive animals appears to reflect the amount of gene transcript expressed per cell rather than the absolute number of cells expressing the *aplnr* gene. We do not know the extent to which changes in *aplnr* expression are mirrored at the protein, and thus functional, level however in previous studies where we have silenced *aplnr* expression in the RVLM we have seen a corresponding loss of APJ functionality [65]. Our data provide a foundation upon which to address the role of APJ in the sensory CVOs, and in the SFO in particular, in cardiovascular control. This finding is in accord with our previous study where we have shown attenuated *c-fos* mRNA expression in the SFO of *aplnr*-KO mice after osmotic stress [66], suggesting reduced neuronal activity in this region in the absence of APJ, and reflecting a role for APJ in the SFO in fluid regulation. We postulate that circulating (and/or local) apelin activates APJ neurons in the SFO, and this increased neuronal activity is relayed downstream directly or indirectly (perhaps via another receptor system such as vasopressin V1a receptors, as shown in the RVLM [30]) to primary SFO projection regions, namely the PVN, RVLM and/or NTS. How the apelinergic system in the CVOs coordinates cardiovascular activity between each CVO, and in line with apelinergic activity in the RVLM/PVN, is not known.

APJ-ir has been localised to both neuronal and glial cell populations within the spinal cord and cortex [16] but no study has reported the APJ-expressing cellular phenotype in the sensory CVOs. In the SFO, OVLT, and AP, *aplnr* and *apln* were expressed in both neurons and glial cells in both SHRs and WKY rats. *Aplnr* and *apln* were not expressed in every neuron or glia, raising the possibility that components of the apelinergic system are present in distinct cell subpopulations. Defining the expression of genes such as those encoding GPCRs and their peptide ligands by transcriptomics may help characterise neuronal and glial cell diversity in CVOs, as has been shown in other brain regions (e.g., [67]). In addition, we report that the expression of neuronal *aplnr* and *apln* is decreased in the AP of SHRs, with an accompanying decrease in the co-localization of *aplnr* with *apln*, in comparison to WKY rats, while the glial expression of receptor and ligand genes is increased in the OVLT of SHRs. As the composition of the CVO neuronal and glial population does not appear to differ between SHRs and WKY rats, changes seen in the expression pattern of *apln* and/or *aplnr* in these CVO cell types may reflect a functional change in activity within the CVOs, indicating a potential dysfunction in the communication between CVOs and downstream signalling pathways in the SHR that may be responsible, at least in part, for the hypertensive phenotype. Our results suggest that the mechanisms by which apelin mediates its hypertensive actions may involve an interaction between the activation of APJ on both neurons and glia within individual CVOs to impact neurophysiology.

Along with its expression in neurons and glia, it is possible that *aplnr* is localised in endothelial cells in the CVOs, which are highly vascularized [68]. Ir-APJ is found in vascular and tissue endothelial cells (ECs) [69, 70] and *aplnr* and *apln* transcripts are present in brain and peripheral tissue ECs [71]. In the brain *aplnr* is present at low levels in normal human meningeal blood vessels [72], and both *apln* and ir-apelin, and *aplnr* expression have been found in microvascular proliferations in human glioblastoma specimens [72, 73]. *Aplnr* is expressed in the ECs of some vessels in the rodent brain [74], and apelin promotes angiogenesis and endothelial function after brain insults in mice [75, 76], and stimulates the proliferation of mouse brain microvasculature-derived ECs [77]. The potential expression of components of the apelinergic system in brain ECs, e.g. using an EC-specific marker such as CD31, warrants investigation in future studies.

In conclusion our study supports the potential physiological importance of APJ and apelin in the sensory CVOs by showing that (1) the SFO, OVLT, and AP, which are linked to the central networks that modulate central cardiovascular responses, express components of the apelinergic system, and (2) the expression of *aplnr* in the SFO is altered in SHRs indicating CVO apelinergic dysfunction in this model of hypertension.
